# Comparative Evaluation of Anticariogenic Activity of Aqueous Extracts of Green Tea and Pomegranate Peel on Bacteria-Induced Secondary Caries at the Tooth-Resin Interface Using a Dye Penetration Method

**DOI:** 10.7759/cureus.99121

**Published:** 2025-12-13

**Authors:** Sangeetha Saji, Praveena G, Drisya Soman, Saranya GS, Nisha B Kurup, Amal S

**Affiliations:** 1 Conservative Dentistry and Endodontics, Azeezia College of Dental Sciences and Research, Kollam, IND

**Keywords:** anticariogenic activity, collagen cross linkers, confocal microscope, green tea, pomegranate peel, resin-dentin interface

## Abstract

Objective

To evaluate and compare the anticariogenic efficacy of aqueous extracts of green tea and pomegranate peel at the resin-dentin interface of human premolars subjected to Streptococcus mutans-induced secondary caries.

Methodology

Twenty-one human premolars were sectioned into 42 specimens, with Class V preparations (3mm x 3mm x 1mm) etched with 37% phosphoric acid. Specimens were divided into three groups: Group I (Control, n=14): Two coats of bonding agent applied and light-cured; Group II (Green Tea): 50% green tea extract applied, followed by bonding agent; Group III (Pomegranate): 30% pomegranate peel extract applied, followed by bonding agent. All specimens were restored with composite in two vertical increments, light-cured for 40 s, and polished. The surfaces were coated with acid-resistant nail varnish, leaving a 1 mm margin around the restoration. After disinfection in 70% ethanol and rinsing twice with sterile phosphate-buffered saline (PBS), specimens were stored in PBS at 4 °C overnight. They were then inoculated with *Streptococcus mutans* (MTCC 890) in brain heart infusion (BHI) broth containing 1% sucrose for five days to induce artificial secondary caries. Following the cariogenic challenge, specimens were rinsed, sectioned longitudinally through the restoration, hydrated, and stained overnight with 0.1 mM Rhodamine B. Confocal microscopy images were obtained at 10× and 60× magnification. Rhodamine B-infiltrated dentin indicated lesion progression, while a dye-free zone near the resin-dentin interface in the experimental groups represented the inhibition zone. The depth of this inhibition zone was measured from the resin-dentin interface to the Rhodamine B-stained region in micrometers.

Results

An independent t-test was used for statistical analysis. In the control group, complete Rhodamine B dye infiltration was observed at the resin-dentin interface, with no inhibition zone detected in any of the 14 samples. In contrast, the experimental groups exhibited a distinct dark zone (inhibition zone) devoid of Rhodamine B dye along the resin-dentin interface. The difference between the green tea group (Group II) and pomegranate group (Group III) was found to be non-significant (p=0.052).

Conclusion

Proanthocyanidin-rich natural collagen crosslinkers, namely pomegranate peel and green tea extracts, can inhibit secondary caries at the resin-dentin interface. As secondary caries is the principal reason for replacement of resin composite restorations, the biomodification of dentin with these natural collagen crosslinkers and matrix metalloproteinase (MMP) inhibitors can stabilize the collagen and protect the resin dentin interface from degradation, thereby decreasing the chances of secondary caries.

## Introduction

Composite restorations have largely supplanted traditional restorative materials due to their superior esthetics and the ability to preserve more natural tooth structure through minimally invasive preparations. However, secondary caries remain the leading cause for the failure and replacement of resin composite restorations [1]. These lesions typically develop at the resin-tooth interface, where bacterial biofilm accumulates in the presence of marginal gaps or imperfections in the seal between the composite and tooth. A key contributing factor is the disparity between the depth of the demineralized collagen layer and the extent of resin penetration. This mismatch leaves collagen fibrils at the base of the hybrid layer partially unprotected by polymerized resin, rendering them susceptible to enzymatic degradation and bacterial invasion.

The absence of resin protection and water in the hybrid layer exposes collagen fibrils at its base to degradation by host-derived matrix metalloproteinases (MMPs) and cysteine cathepsins [2]. MMPs are naturally occurring proteolytic enzymes that are activated during acid etching and dentin bonding. Once triggered, they gradually degrade the collagen fibrils within the hybrid layer, leading to microleakage and promoting secondary caries development. In addition to MMPs, cysteine cathepsins significantly contribute to dentin breakdown by degrading its organic matrix.

Over time, the bond between the tooth structure and restorative materials weakens due to hydrolytic or mechanical deterioration of the hybrid layer [3]. This degradation compromises the seal at the tooth-restoration interface. Therefore, maintaining a stable and organic seal is essential for long-term adhesion. Streptococcus mutans is the primary pathogenic microorganism responsible for the initiation and progression of dental caries [4].

Recently, the concept of dentin biomodification has emerged as a strategy to achieve a more stable and long-lasting adhesive interface [5]. This approach involves the use of various natural and synthetic agents that function as MMP inhibitors and collagen cross-linkers, thereby enhancing the mechanical properties of the dentin substrate [6]. Studies have demonstrated the specific anticariogenic effects of proanthocyanidin-rich agents, such as pomegranate peel and green tea, against Streptococcus mutans. These agents disrupt polysaccharide synthesis, thereby interfering with the bacteria's adherence mechanisms to both tooth surfaces and restorative materials. As a result, their anticariogenic potential can be explored at the resin-dentin interface to help prevent the failure of composite restorations. Effective suppression of Streptococcus mutans and other cariogenic organisms at restoration margins, as reported for orthodontic bonding materials (Alam et al., 2024 [7]), supports the rationale for testing natural bioactive agents such as green tea and pomegranate peel extracts.

Published evidence on the impact of other proanthocyanidin-rich agents, such as green tea and pomegranate peel extracts, on the resin-dentin interface remains limited. Given that these naturally derived agents have demonstrated a broad spectrum of anticariogenic activities, it is important to investigate and compare their protective effects on the highly susceptible resin-dentin interface. Therefore, the present study aims to evaluate and compare the anticariogenic efficacy of aqueous extracts of green tea and pomegranate peel against bacteria-induced secondary caries at the resin-dentin interface using confocal microscopy.

Rationale

Pre-treatment of demineralized dentin with proanthocyanidin-rich extracts (e.g., from grape seed) simultaneously enhances collagen matrix stiffness and cross-linking and inhibits up to ~70-90 % of MMP/cysteine cathepsin activity, thus stabilising the resin-dentin interface and reducing endogenous proteolytic degradation [5,8].

## Materials and methods

Tooth selection

Twenty-one freshly extracted human premolars (extracted for orthodontic reasons) were collected for the study after obtaining informed consent from patients as per the protocol of the institutional ethics committee (AEC/REV/2017/48). Only teeth free from dental caries, cracks, restorations or developmental defects were selected for this study. The teeth were stored in 0.1% thymol until use.

Restorative procedures and specimen preparation

Each tooth was first cleaned using an ultrasonic scaler to remove debris. The root portion was sectioned 1 mm below the cementoenamel junction (CEJ) with a diamond disc. The coronal portion was then sectioned mesiodistally along the long axis into buccal and lingual halves, yielding a total of 42 halves. Standardized Class V cavities measuring 3 mm × 3 mm × 1 mm were prepared on all specimens using a No. 245 carbide bur (3 mm length, 0.8 mm diameter) in a high-speed handpiece with air/water coolant. Cavity preparation was initiated with a punch cut placed 1 mm above the CEJ on the mid-labial aspect of the cervical third. The preparation was extended mesiodistally to remain within the 3 mm × 3 mm dimension, and cavity depth was standardized at 1 mm by marking the bur. The same procedure was repeated for all specimens. Cavity walls were etched with 37% phosphoric acid for 15 seconds, rinsed with distilled water for 30 seconds, and gently blotted dry with absorbent tissue. The prepared cavities were then randomly divided into three groups based on the surface treatment applied to the acid-etched dentin.

Preparation of aqueous green tea extract

Ten grams of green tea was weighed using an electronic balance. One hundred milliliters of distilled water was measured with a graduated cylinder and transferred into a round-bottom flask. The flask was placed on a heating mantle, and the temperature was set to 100 °C. The 10 g of green tea was then added to the boiling distilled water, and the mixture was boiled for 30 minutes. The resulting extract was concentrated to 10 ml, yielding a 100% (w/v) solution. This extract was subsequently diluted with distilled water in a 1:1 ratio to obtain a 50% (w/v) aqueous extract.

Preparation of aqueous pomegranate peel extract

**Figure 1:**
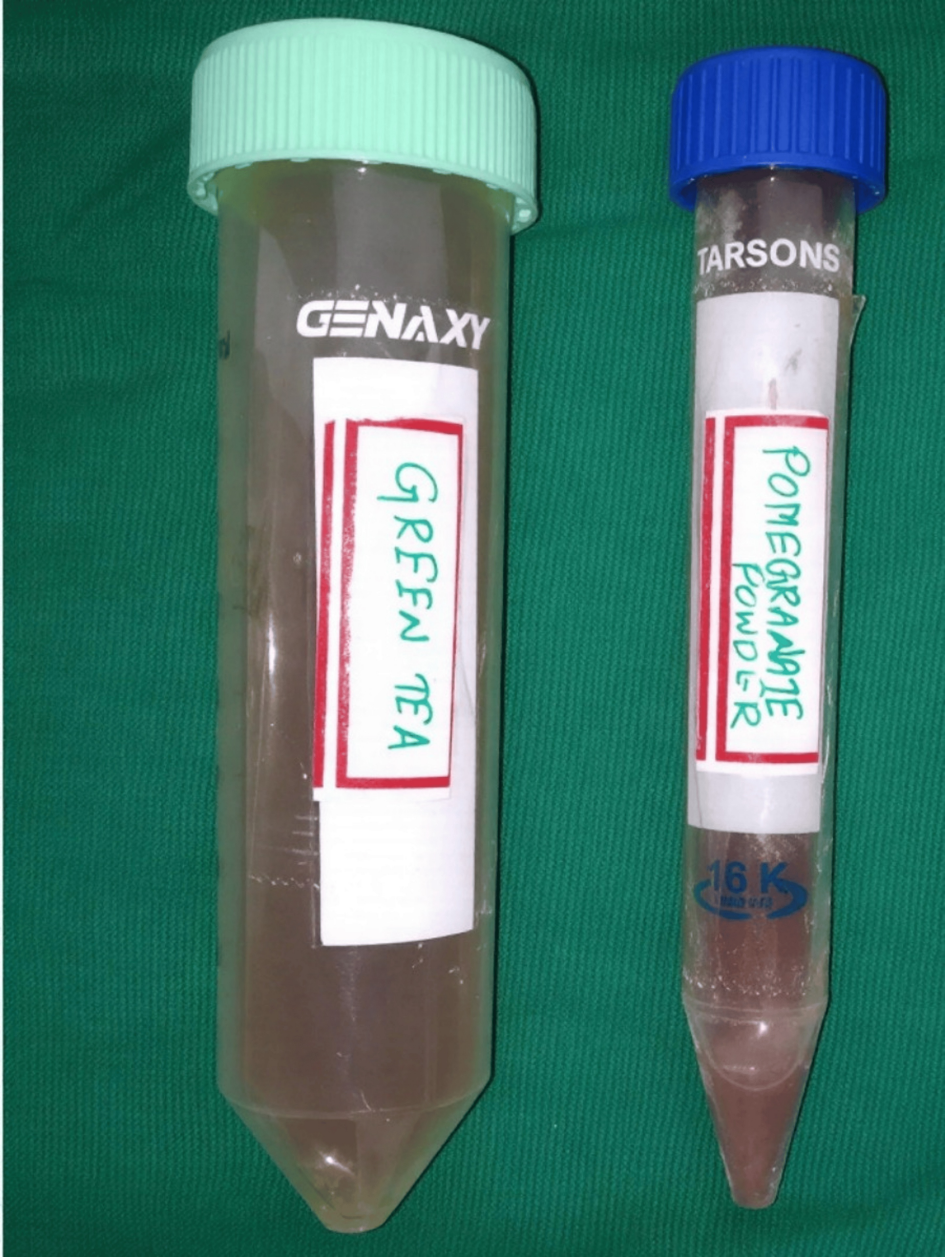
Aqueous Extracts of Green Tea and Pomegranate Peel

Dried pomegranate peel was ground into a fine powder using a heavy-duty kitchen grinder. From this, 18 g of powder was weighed on an electronic balance. Sixty milliliters of distilled water was measured with a graduated cylinder and boiled in a heating mantle at 100 °C. The pomegranate peel powder was then added to the boiling water, and the mixture was filtered through filter paper. The filtrate collected represented a 30% aqueous pomegranate peel extract. Both extracts were stored separately in labeled containers until use (Figure 1).

Restorative procedures and specimen preparation

Teeth were cleaned with an ultrasonic scaler and sectioned 1 mm below the CEJ using a diamond disc. The coronal portion was split mesiodistally into buccal and lingual halves, yielding 42 specimens. Standardized Class V cavities (3 × 3 × 1 mm) were prepared on the mid-labial surface, 1 mm above the CEJ, with a No. 245 carbide bur in a high-speed handpiece under air-water spray. The cavity depth was standardized to 1 mm by a bur marking. Prepared cavities were etched with 37% phosphoric acid for 15 seconds, rinsed with distilled water for 30 seconds, and blotted dry. Specimens were then randomly divided into three groups according to the dentin surface treatment: Group I (Control Group) - bonding agent + composite; Group II (Green Tea Group) - green tea extract + bonding agent + composite; and Group III (Pomegranate Group) - pomegranate peel extract + bonding agent + composite.

Group I (Control, n=14)

Two coats of bonding agent were applied to the prepared cavity surface using a micro-applicator and light-cured for 20 seconds with an LED unit. Composite resin was then placed in two increments - an occlusal increment filling the occlusal half and a gingival increment sealing the gingival margin - each cured for 20 seconds. The restorations were finished and polished using sequential coarse, medium, and fine grit Al₂O₃ abrasive discs at slow speed.

Group II (Green Tea Group)

A 50% aqueous green tea extract was dispensed into a dappen dish and applied to acid-etched dentin using a microbrush for 60 seconds. Excess was blotted with absorbent tissue. Two coats of bonding agent were applied with a micro-applicator, followed by light curing for 20 seconds using an LED unit. Composite resin was placed in two increments: the occlusal half was filled and cured for 20 seconds, followed by the gingival half to seal the margin, also cured for 20 seconds. Restorations were polished with coarse, medium, and fine Sof-Lex (3M ESPE, Seefeld, Germany) Al₂O₃ discs in a slow-speed handpiece.

Group III (Pomegranate Group)

A 30% aqueous pomegranate peel extract was dispensed into a dappen dish and applied to acid-etched dentin with a microbrush for 60 seconds. After blotting excess, two coats of bonding agent were applied with a micro-applicator and light-cured for 20 seconds. Composite resin was placed incrementally: the occlusal portion was filled and cured for 20 seconds, followed by the gingival portion to seal the margin, also cured for 20 seconds. Final polishing was performed with coarse, medium, and fine Sof-Lex Al₂O₃ discs in a slow-speed handpiece.

Artificially induced secondary caries

Restored specimens were coated with acid-resistant nail varnish, leaving a 1 mm margin around the restoration exposed for demineralization challenge. After air-drying for 30 minutes, specimens were disinfected in 70% ethanol, rinsed twice with sterile phosphate-buffered saline (PBS), and stored overnight at 4°C in PBS. They were then inoculated with Streptococcus mutans suspension (MTCC 890) in brain heart infusion broth supplemented with 1% sucrose for five days, with the medium refreshed on day three [9]. Following incubation, specimens were rinsed thoroughly with water, sectioned longitudinally through the restoration, hydrated in distilled water for one hour, and stained overnight with 0.1 mM Rhodamine B solution (pH 7.2).

Sample processing and imaging

After rinsing and blot drying, one-half of each sectioned tooth was analyzed under a confocal laser scanning microscope equipped with a digital camera [10]. Both differential interference contrast (DIC) and red fluorescence images (excitation 529 nm) were obtained using standardized settings. Image analysis was carried out with Nis Elements software (Nikon Instruments Inc., Tokyo, Japan) at 10× and 60× magnification to assess dye penetration in the dentin adjacent to restoration margins for both control and experimental groups. In the experimental groups, a dark zone with minimal dye penetration was observed near the resin-dentin interface. This dye-free region was considered an inhibition zone against secondary caries. Images were recorded at 10× and 60× magnifications, and the depth of the inhibition zone was measured in micrometers at 60× for each specimen. The values were then tabulated.

## Results

Data were entered into a Microsoft Excel (Redmond, WA, USA) spreadsheet and analyzed using SPSS for Windows (Statistical Package for the Social Sciences, SPSS Inc., Chicago, IL, USA), version 17.0. Continuous variables were expressed as mean ± standard deviation. An independent t-test was applied to compare the groups. A p-value of < 0.05 was considered statistically significant, following the assumptions of standard statistical tests (Table 1).

**Table 1:** Showing Mean Depth of Inhibition Zone in All Groups Tested

Group	N	MEAN(µm)	STD. DEVIATION	STD. ERROR MEAN	P-VALUE
Group 1 Control	14	0			.052
Group II Green Tea Group	14	17.00230	5.826661	1.842552	
Group III Pomegranate Group	14	24.18000	16.223710	5.130388	

**Figure 2:**
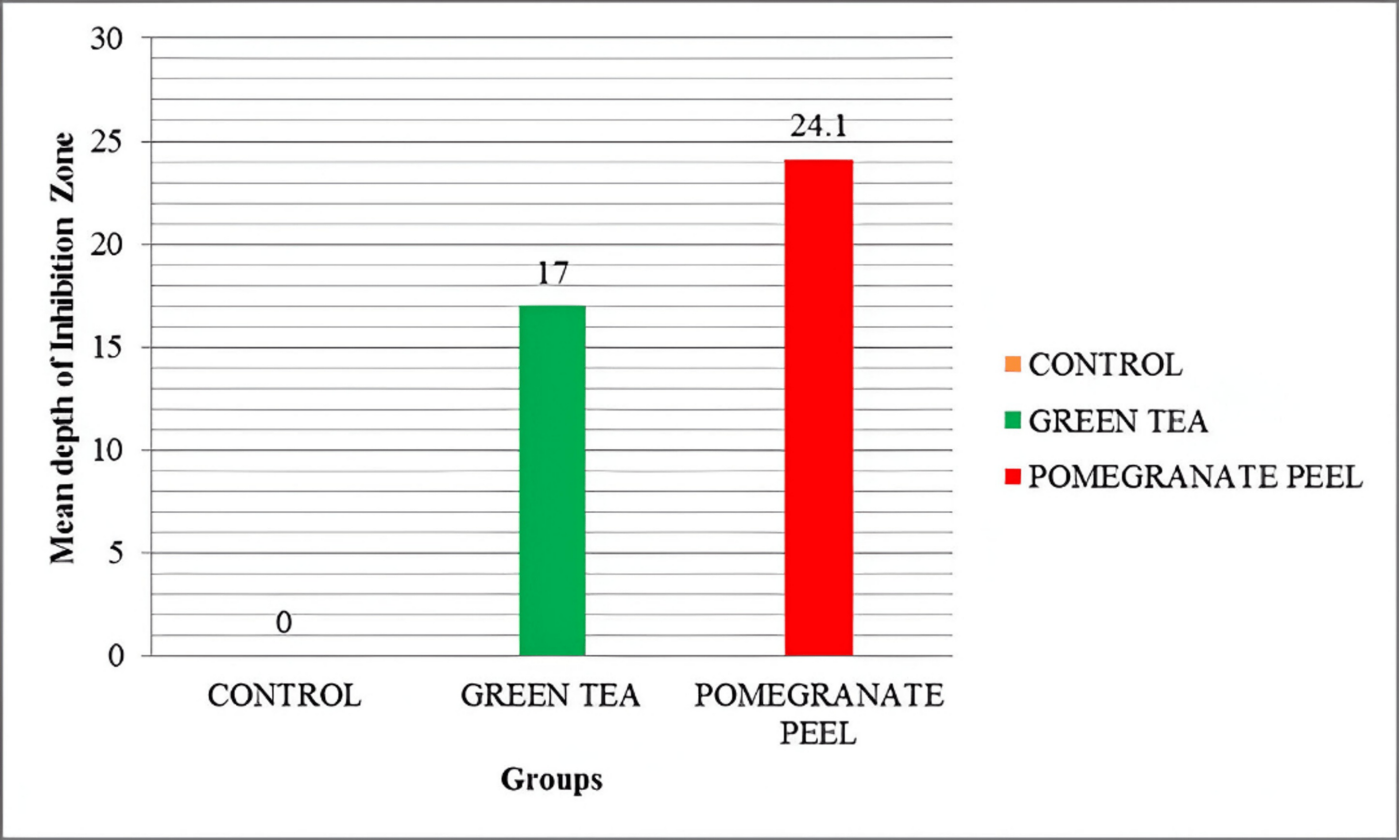
Bar Graph Showing Mean Depth of Inhibition Zone in Three Groups

A bar graph showing the mean depth of the inhibition zone in three groups is shown below (Figure 2).

**Figure 3:**
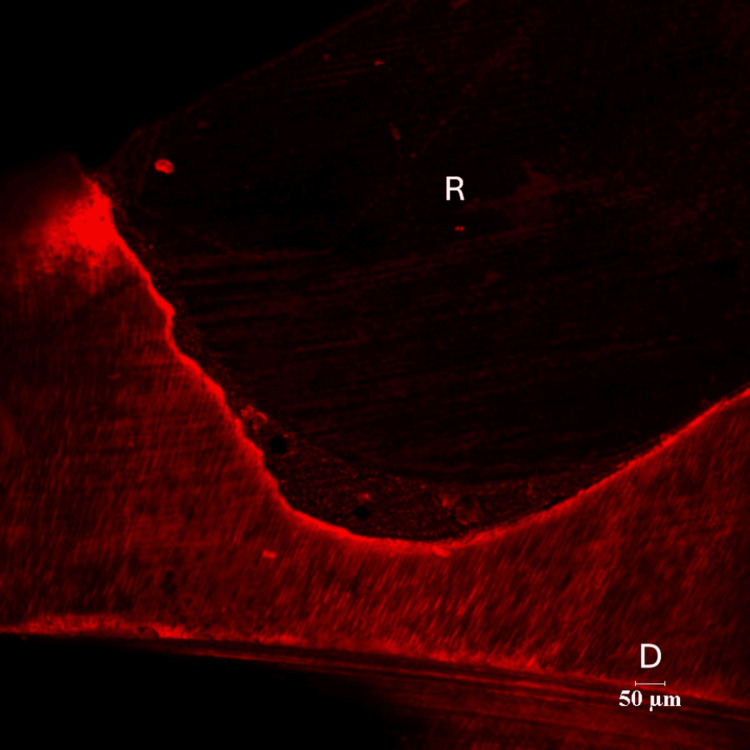
Representative Images of Rhodamine B-Infiltrated Dentin-Control Group (10 x Magnification) R - Resin D- Dentin

**Figure 4:**
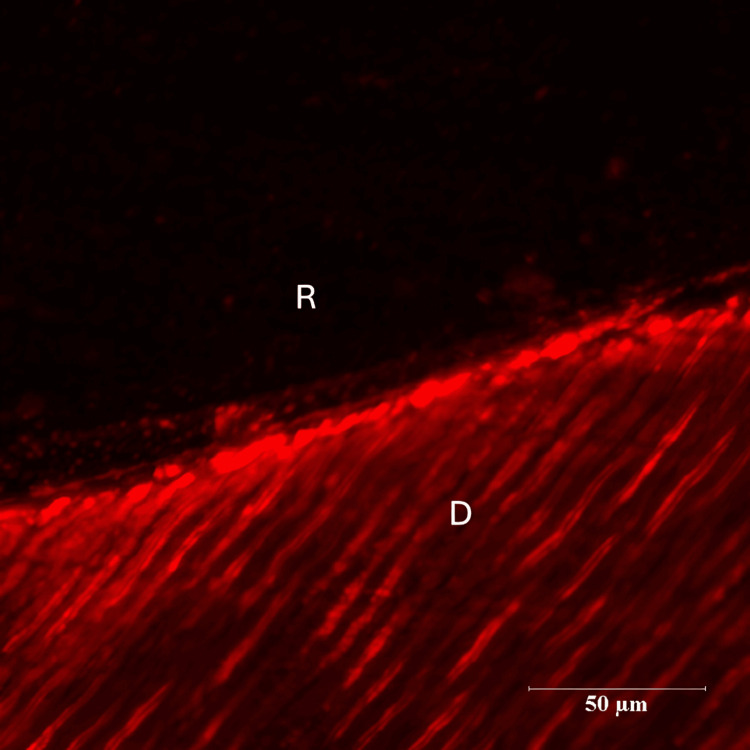
Representative Images of Rhodamine B-Infiltrated Dentin-Control Group (60x Magnification) R- Resin D-Dentin

There were three groups in the study - Control group (Group I), Green tea group (Group II) and Pomegranate group (Group III). Each group consisted of 14 specimens. Positions of the resin-dentin interface were identified in confocal microscopic images. In the control group, there was a complete infiltration of the Rhodamine B dye at the resin-dentin interface and no inhibition zone was seen (Figures 3, 4).

**Figure 5:**
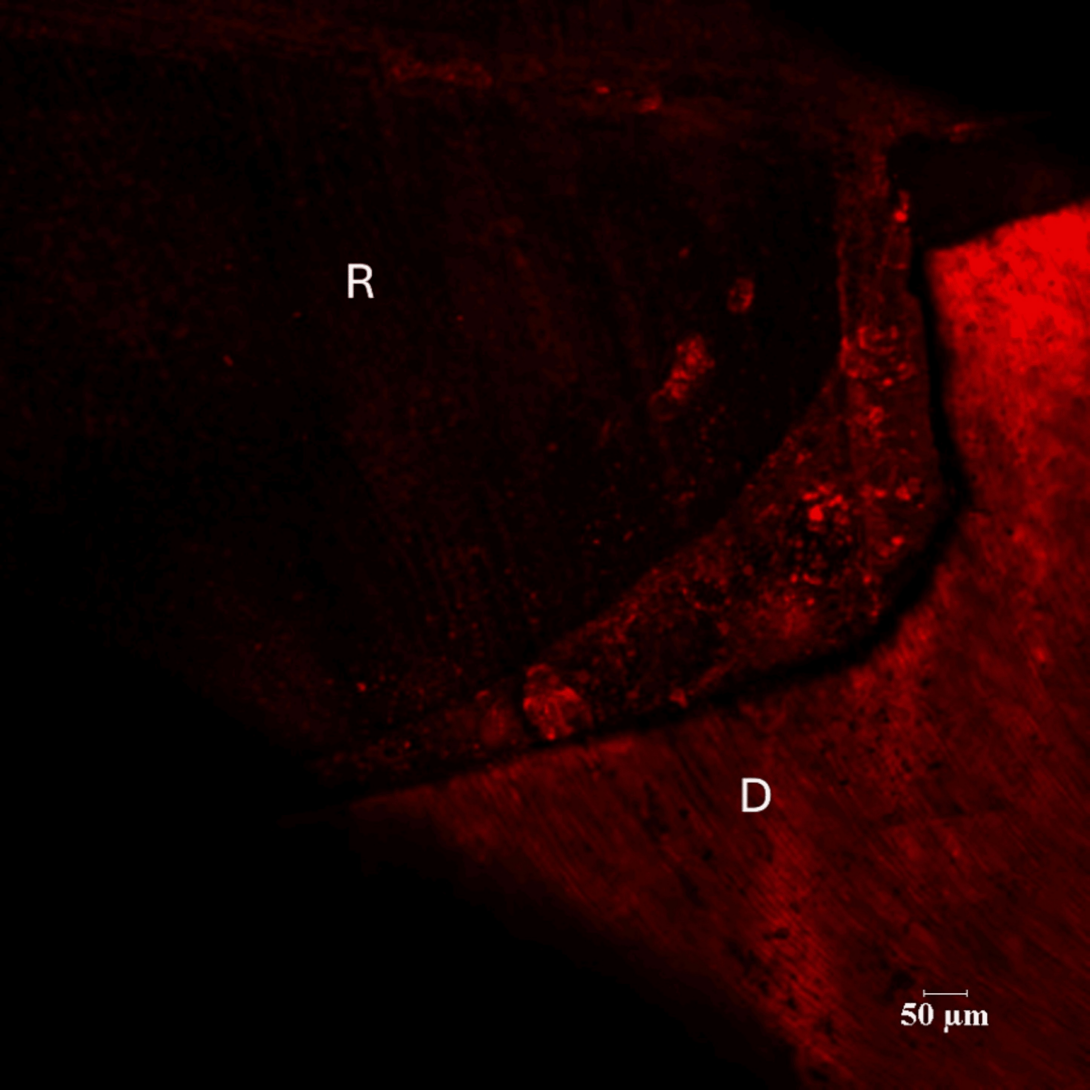
Green Tea Group (10X Magnification) R- Resin D- Dentin

**Figure 6:**
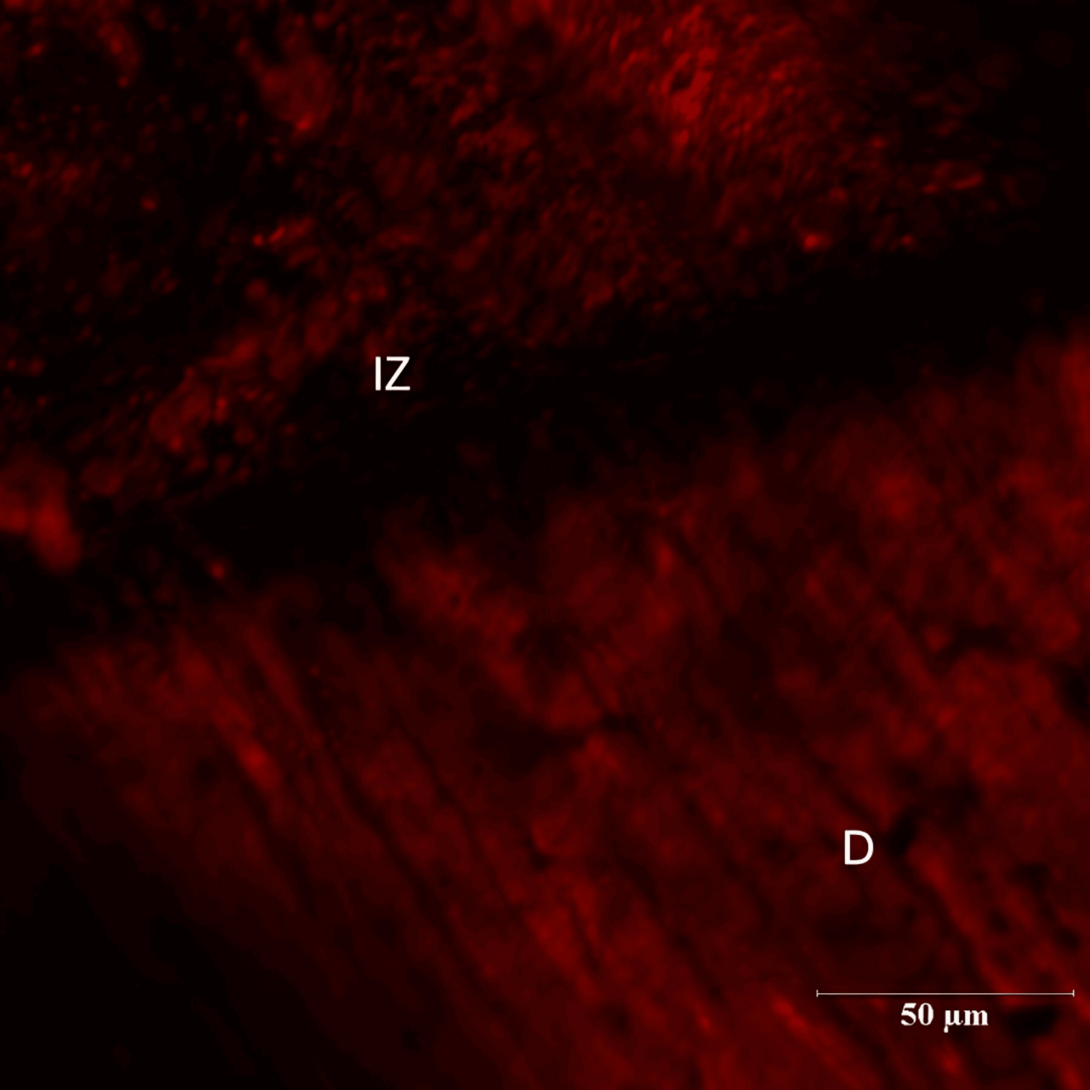
Green Tea Group (60X Magnification) IZ-Inhibition Zone D- Dentin

In the experimental groups a dark zone devoid of the Rhodamine B dye was seen towards the dentin throughout the resin-dentin interface. Depth of inhibition zone was measured from the resin-dentin interface to the area of Rhodamine B stained in µm. The control group could not produce an inhibition zone in all 14 samples. Among 14 samples of green tea group (Group II), the maximum value of inhibition zone was 27.45 µm whereas the least value obtained was 5.60 µm. The mean depth of inhibition zone was derived as 17.00230±5.826661 for the green tea group (Figures 5, 6).

**Figure 7:**
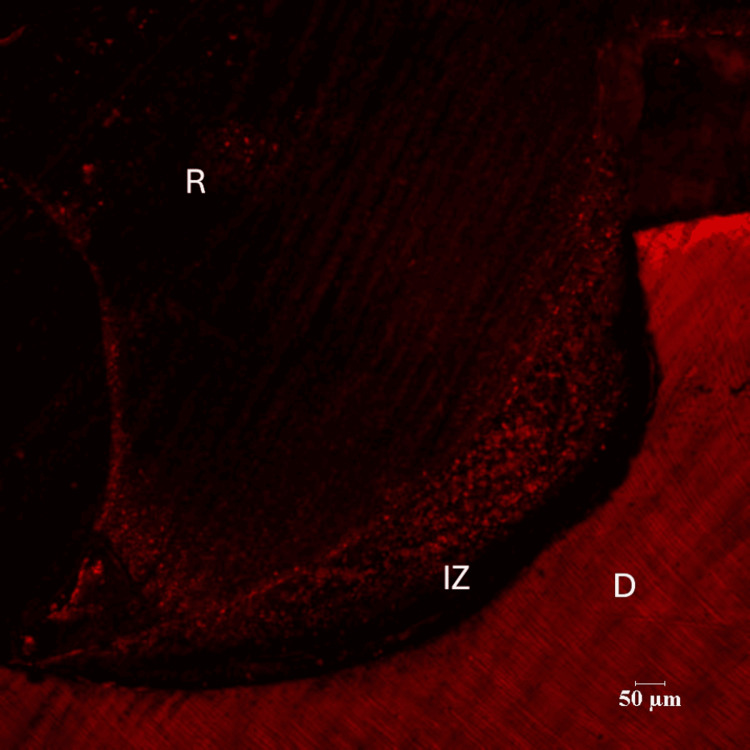
Pomegranate Group (10X Magnification) R-Resin D-Dentin IZ-Inhibition Zone

**Figure 8:**
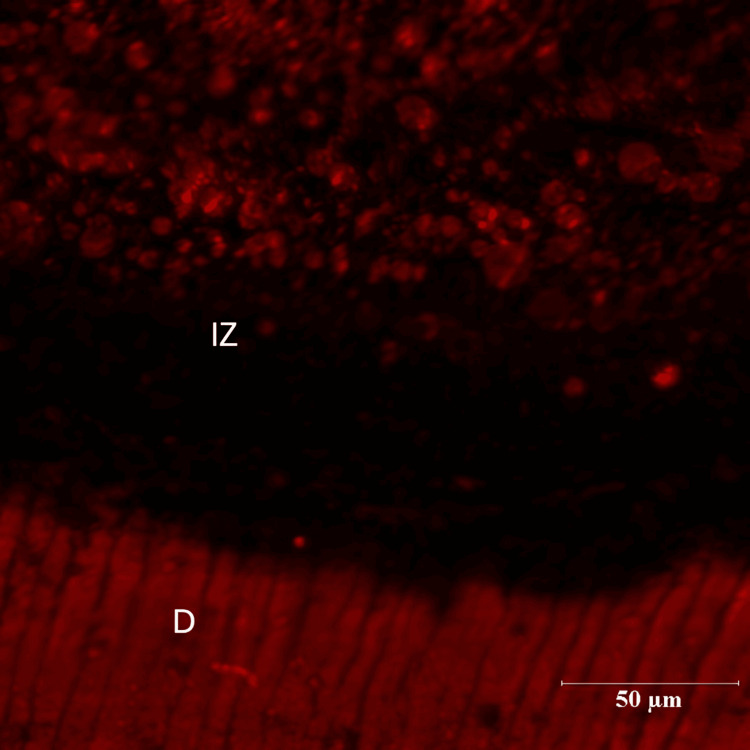
Pomegranate Group (60X Magnification) D-Dentin IZ-Inhibition Zone

In the pomegranate group (Group III), the maximum depth of inhibition zone was 64.84 µm while the least value obtained was 13.01 µm. The mean depth of inhibition zone was 24.18000±16.223710 for the pomegranate group (n=14) (Figures 7, 8). The difference between the green tea group (Group II) and pomegranate group (Group III) was found to be non-significant (p=.052).

## Discussion

Preserving the integrity of the resin-dentin interface remains a clinical challenge, as dentin is a heterogeneous substrate characterized by its high protein content and inherent moisture. The stability of this interface plays a crucial role in influencing the progression of caries.

The formation of a durable resin-dentin bond is hindered by several factors, including water sorption and hydrolysis of the adhesive resin, insufficient monomer-to-polymer conversion of the infiltrating adhesive, incomplete resin infiltration, and inadequate solvent evaporation [11,12]. To address these challenges, various strategies have been proposed, such as minimizing the depth of dentin demineralization, using rubber dam isolation to prevent contamination by moisture or saliva, ensuring proper handling of composite materials, and carefully managing and storing adhesives to reduce solvent loss [13].

Proanthocyanidin (PA) is a polyphenol-rich bioflavonoid, structurally based on flavanols, and is commonly found in a variety of fruits, vegetables, nuts, seeds, and flowers [14]. PAs are well known for their broad-spectrum antimicrobial properties. Specifically against cariogenic bacteria, PAs inhibit surface-adsorbed glucosyltransferases (GTFs) and reduce acid production by Streptococcus mutans, while also suppressing bacterial growth and biofilm formation [15]. These antimicrobial effects help prevent dentin demineralization, as dentin can serve as a reservoir for PAs bound to the collagen matrix. Beyond their antimicrobial activity, PAs facilitate dentin biomodification by promoting collagen cross-linking and inhibiting MMPs. These effects improve the mechanical stability of the resin-dentin interface by strengthening the dentin matrix, maintaining collagen integrity, and supporting the formation of a more durable hybrid layer during adhesive bonding.

Natural collagen cross-linkers include proanthocyanidin-rich agents such as grape seed extract, epigallocatechin-3-gallate (EGCG) from green tea, punicalagin from pomegranate peel, hesperidin (HPN), genipin (GNP), and riboflavin. Proanthocyanidins reduce MMP activity by stiffening collagen polypeptides, thereby preventing their unwinding. Additionally, they can inactivate the catalytic sites of host-derived MMPs by forming new peptide bonds between adjacent collagen chains. A key advantage of these naturally derived cross-linkers is their excellent biocompatibility [16]. Given the high cytotoxicity associated with synthetic cross-linkers, this study employed proanthocyanidins containing various polyphenols - specifically epigallocatechin-3-gallate from green tea and punicalagin from pomegranate peel extracts.

There are two methods to provide an artificial cariogenic challenge to the tooth structure: purely chemical systems which use an acidic environment to demineralize the tooth [17] and bacterial systems in which a specific bacterial culture causes demineralization [18]. In this study, specimens were inoculated with Streptococcus mutans for a period of five days. The duration was chosen based on the findings of Fontana et al. (1996), who reported that a mixture of overnight cultures of Streptococcus mutans and Lactobacillus casei was able to induce secondary carious lesions within seven days [19]. These lesions exhibited surface and wall characteristics consistent with those observed in vivo, as described by Kidd et al. [20]. Similarly, Kim et al. (2017) incubated Class V restorations on bovine incisors with Streptococcus mutans in a bacterial model for four days [21].

Confocal laser scanning microscopy was used in this study due to its noninvasive and nondestructive imaging capabilities [22]. In dentistry, confocal microscopy has a wide range of applications, including caries research, evaluation of soft and hard tissue responses to biomaterials and implants, and assessment of the sealer-dentin interface, as well as the presence and distribution of sealers or adhesives within dentinal tubules [23]. Specific pathogenic oral bacteria can also be fluorescently tagged with antibodies, allowing for simultaneous visualization in carious lesions. Additionally, fluorescent dyes can be incorporated into dentin bonding agents or glass ionomer components for enhanced imaging [24].

Confocal microscopy offers several advantages over conventional techniques, including the elimination of radiation exposure and a faster, more straightforward analysis compared to microradiography. In the present study, a 0.1 mM Rhodamine B solution was selected due to its ability to penetrate the fine porosities of carious lesions, providing uniform staining. Extracted human teeth with secondary carious lesions were sectioned through the restorations, stained with Rhodamine B, and examined under the confocal system. Dye infiltration into demineralized regions enabled a quantitative evaluation of lesion porosity.

There were three groups in the study: Control group (Group I), Green tea group (Group II) and Pomegranate group (Group III). Each group consisted of 14 specimens. Positions of the resin-dentin interface were identified in confocal microscopic images. In the control group, there was a complete infiltration of Rhodamine B dye at the resin-dentin interface and no inhibition zone was seen. In the experimental groups, a dark zone devoid of the Rhodamine B dye was seen towards the dentin throughout the resin-dentin interface. This dye-free zone was assumed to be an inhibition zone against secondary caries. Depth of the inhibition zone was measured from the resin-dentin interface to the area of Rhodamine B stained in µm. The control group could not produce an inhibition zone in all 14 samples, which implies secondary caries development along resin-dentin interface. Among 14 samples of green tea group (Group II), the mean depth of inhibition zone was derived as 17.00230±5.826661. In the pomegranate group (Group III), the mean depth of inhibition zone was 24.18000±16.223710 (n=14). The difference between the green tea group (Group II) and pomegranate group (Group III) was found to be non-significant (p=.052). In this study aqueous extracts of green tea and pomegranate peel extracts inhibited secondary caries development adjacent to the resin-dentin interface compared to the control group. Hence the research hypothesis in the present study can be accepted.

The pomegranate group exhibited a larger zone of inhibition compared to the green tea group, although the difference was not statistically significant (p = 0.052). A possible explanation for this observation is the presence of punicalagin, a predominant polyphenol in pomegranate peel that accounts for over 50% of its antioxidant activity. Punicalagin has been reported to be more potent than EGCG - the primary catechin in green tea - in inhibiting MMP activity. Pomegranate polyphenols enhance resistance to collagenase degradation by forming hydrogen bonds with free amino acids in collagen fibers. Additionally, hydrophobic and electrostatic interactions may further contribute to collagen stabilization [25]. The findings of this study support the strong antibacterial activity of aqueous pomegranate peel extracts against Streptococcus mutans, aligning with previous research by Ferrazzano GF et al. (2017), who reported potent antibacterial effects of pomegranate peel against both Gram-positive and Gram-negative bacteria [26].

Studies have also demonstrated the specific antimicrobial mechanism of pomegranate peel against dental biofilm bacteria, including interference with polysaccharide synthesis, thereby disrupting bacterial adherence to tooth surfaces [27]. Increasing interest has been directed toward the use of tannins for caries prevention. Tannins can penetrate bacterial cell walls, which are composed of proteins and polysaccharides, and bind to their surfaces. They have the ability to precipitate proteins and inhibit enzymes such as glucosyltransferases. The hydrolysable tannins in pomegranate can form high-molecular-weight complexes with soluble proteins, promote bacterial lysis, and impair bacterial adhesion [28].

Umar D et al. (2016) conducted an in vivo study to evaluate the effects of pomegranate-based mouth rinses - including peel extract, aril extract, and juice - on Streptococcus mutans (MTCC 890) counts and salivary pH in healthy individuals [29]. These effects were assessed before and after mouth rinsing and compared with those of 0.2% chlorhexidine. The study concluded that pomegranate peel extract, at concentrations of 300 mg/mL and 600 mg/mL, effectively reduced S. mutans counts and increased salivary pH.

Green tea is a rich source of PAs, particularly the monomeric compound EGCG. These PAs have been shown to enhance the durability of the resin-dentin bond, particularly in caries-affected dentin where MMPs and cysteine cathepsins are highly active [30]. The application of EGCG has demonstrated the ability to stabilize dentin collagen fibrils, increasing their resistance to enzymatic degradation [8]. Monomeric catechins and epicatechins - key components of PAs - possess phenolic hydroxyl groups that enable multiple hydrogen bonding interactions with proteins. Galloylated catechins, such as EGCG, have an even higher hydrogen bonding capacity due to the presence of three vicinal hydroxyl groups in the galloyl moiety [31]. EGCG also promotes collagen cross-linking, stabilizing the triple-helical structure and strengthening the fibrillar matrix [32]. Additionally, it impedes the binding of collagenases to active sites on collagen chains, thereby helping to prevent collagen degradation [33].

In a 2012 study, Subramaniam P et al. examined the antibacterial effects of aqueous and organic extracts of three types of tea-green, black, and oolong-on Streptococcus mutans. The extracts were inoculated into wells on Mueller-Hinton agar plates pre-inoculated with S. mutans. Aqueous extracts of green tea and oolong tea, at 50% and 100% concentrations, produced larger zones of inhibition than chlorhexidine [34]. Similarly, Anita et al. (2015) evaluated the in vitro antimicrobial activity of green tea extract against Streptococcus mutans (MTCC 890) and Lactobacillus acidophilus, concluding that green tea exhibits notable antibacterial activity against these key cariogenic bacteria [35]. In 2013, Fonseca et al. reported that pre-treatment of acid-demineralized dentin collagen with aqueous green tea extracts did not adversely affect the immediate microtensile bond strength of a two-step etch-and-rinse adhesive system [36].

This study employed an in vitro microbial-based artificial model to induce incipient secondary carious lesions and to assess the depth of inhibition zones produced by anticariogenic agents, using a reproducible and quantifiable technique such as confocal microscopy. Despite its advantages, the bacterial-induced secondary caries model has limitations when compared to in vivo conditions. Only a single cariogenic species (Streptococcus mutans) was used, and although this permitted lesion formation, the model did not simulate the complex oral environment, where saliva, antimicrobial proteins, and enzymes play important roles in caries development.

In addition, a PBS solution was employed as the mineral wash, rather than glycoprotein-enriched artificial saliva, to maintain controlled bacterial nutrient sources. The model also did not account for specimen aging, which may influence long-term outcomes. Another consideration is the dark brown pigmentation of aqueous green tea and pomegranate peel extracts, which can stain dentin and restorative margins. Developing methods to isolate purified bioactive compounds would be necessary to minimize discoloration without compromising their anticariogenic potential.

As with any in vitro investigation, results should be interpreted with caution when extrapolated to clinical practice [37]. Nevertheless, the present design yielded significant and encouraging findings. Incorporation of green tea and pomegranate peel extracts into resin composite restorations may contribute to the prevention of secondary caries at susceptible resin-dentin margins. Further in vitro and in vivo studies are recommended to validate these results and to investigate the clinical applicability of Proanthocyanidin treatment as a collagen cross-linking strategy in restorative dentistry.

Limitations of the study

The present study was conducted under controlled in-vitro conditions, which may not completely replicate the complex oral environment, including variations in saliva composition, pH fluctuations, dietary influences, and microbial diversity. The relatively small sample size and short evaluation period may also limit the generalizability of the findings. Furthermore, only two natural extracts were tested, without comparison to a wider range of available natural or synthetic agents. Hence, clinical trials with larger samples and longer observation periods are required to validate the anticariogenic potential of these agents in vivo.

## Conclusions

Within the limitations of the present study, it may be concluded that proanthocyanidin-rich natural agents such as green tea and pomegranate peel extracts demonstrated an inhibitory effect on the development of secondary caries adjacent to the resin-dentin interface. Both agents contributed to the preservation of the hybrid layer, thereby enhancing the durability of the resin-dentin bond. Among the two, pomegranate peel extract exhibited comparatively better anticariogenic potential, possibly due to its higher concentration of bioactive polyphenols such as punicalagin, which are known for their potent antioxidant and collagen-stabilizing effects. Although the difference in efficacy between pomegranate peel extract and green tea was not statistically significant, the observed trend suggests that pomegranate peel extract may serve as a more promising adjunct in strategies aimed at reducing secondary caries formation. Further long-term studies with larger sample sizes and clinical trials are required to substantiate these findings and to establish the therapeutic potential of these natural agents in restorative dentistry.
